# A gap existed between physicians’ perceptions and performance of pain, agitation-sedation and delirium assessments in Chinese intensive care units

**DOI:** 10.1186/s12871-021-01286-w

**Published:** 2021-02-25

**Authors:** Kai Chen, Yan-Lin Yang, Hong-Liang Li, Dan Xiao, Yang Wang, Linlin Zhang, Jian-Xin Zhou

**Affiliations:** 1 Department of Critical Care Medicine, Beijing Tiantan Hospital, Capital Medical University, No. 119 South Fourth Ring West Road, Fengtai District, Beijing, 100070 China; 2 China National Clinical Research Center for Neurological Diseases, Beijing Tiantan Hospital, Capital Medical University, Beijing, 100070 China; 3 Medical Research & Biometrics Center, National Center for Cardiovascular Disease, Fuwai Hospital, Chinese Academy of Medical Sciences and Peking Union Medical College, Beijing, 100037 China

**Keywords:** Analgesia, Sedation, Practice, Prevalence, Survey, Critical care

## Abstract

**Background:**

Pain, agitation-sedation and delirium management are crucial elements in the care of critically ill patients. In the present study, we aimed to present the current practice of pain, agitation-sedation and delirium assessments in Chinese intensive care units (ICUs) and investigate the gap between physicians’ perception and actual clinical performance.

**Methods:**

We sent invitations to the 33 members of the Neuro-Critical Care Committee affiliated with the Chinese Association of Critical Care Physicians. Finally, 24 ICUs (14 general-, 5 neuroscience-, 3 surgical-, and 2 emergency-ICUs) from 20 hospitals participated in this one-day point prevalence study combined with an on-site questionnaire survey. We enrolled adult ICU admitted patients with a length of stay ≥24 h, who were divided into the brain-injured group or non-brain-injured group. The hospital records and nursing records during the 24-h period prior to enrollment were reviewed. Actual evaluations of pain, agitation-sedation and delirium were documented. We invited physicians on-duty during the 24 h prior to the patients’ enrollment to complete a survey questionnaire, which contained attitude for importance of pain, agitation-sedation and delirium assessments.

**Results:**

We enrolled 387 patients including 261 (67.4%) brain-injured and 126 (32.6%) non-brain-injured patients. There were 19.9% (95% confidence interval [CI]: 15.9–23.9%) and 25.6% (95% CI: 21.2–29.9%) patients receiving the pain and agitation-sedation scale assessment, respectively. The rates of these two types of assessments were significantly lower in brain-injured patients than non-brain-injured patients (*p* = 0.003 and < 0.001). Delirium assessment was only performed in three patients (0.8, 95% CI: 0.1–1.7%). In questionnaires collected from 91 physicians, 70.3% (95% CI: 60.8–79.9%) and 82.4% (95% CI: 74.4–90.4%) reported routine use of pain and agitation-sedation scale assessments, respectively. More than half of the physicians (52.7, 95% CI: 42.3–63.2%) reported daily screening for delirium using an assessment scale.

**Conclusions:**

The actual prevalence of pain, agitation-sedation and delirium assessment, especially delirium screening, was suboptimal in Chinese ICUs. There is a gap between physicians’ perceptions and actual clinical practice in pain, agitation-sedation and delirium assessments. Our results will prompt further quality improvement projects to optimize the practice of pain, agitation-sedation and delirium management in China.

**Trial registration:**

ClinicalTrials.gov, identifier NCT03975751. Retrospectively registered on 2 June 2019.

**Supplementary Information:**

The online version contains supplementary material available at 10.1186/s12871-021-01286-w.

## Background

Pain, agitation-sedation and delirium (PAD) management is one of the key elements in the care of critically ill patients. To date, several guidelines and consensus statements have recommended that the comprehensive evaluation of PAD is the first step in optimizing analgesia and sedation in the intensive care unit (ICU) [1–3]. However, the quality of care may be suboptimal due to the difference between actual practices and evidence-based best practices [4]. International and national investigations revealed that the actual rate of the performance of PAD assessments was markedly lower than the rate perceived by the physicians [5, 6]. In a nationwide survey in China, the rates of PAD assessment were reported as ranging from 67 to 90% [7]. However, a Chinese multicenter cohort study found that the pain and sedation scales were only assessed in approximately 15% of ICU patients [8]. Investigations into the gap between actual clinical practices and physicians’ attitudes are warranted to facilitate quality improvement programs for PAD management in Chinese ICUs.

Critically brain-injured patients pose particular challenges in PAD management [9, 10]. Although consciousness impairment is prevalent in neurological/neurosurgical ICUs [11], PAD can be systematically assessed in critically brain-injured patients [12, 13]. Several consensus statements have recommended strategies for evaluating and treating PAD in acute brain-injured patients [14–16]. However, only scarce data could be found to demonstrate clinical PAD management practices in this population [17–20].

In this study in Chinese ICUs, we primarily aimed to present the current practice regarding PAD assessments, which was compared with the physicians’ perception of the practice obtained from an on-site questionnaire survey. We also deliberately focused on PAD management in ICU-admitted brain-injured patients.

## Methods

### Study design and ethics

The study design was a cross-sectional one-day point prevalence investigation combined with an on-site questionnaire survey. The IRB of Beijing Tiantan Hospital approved the study protocol (KY2017–062-02), which was registered at ClinicalTrials.gov (NCT03975751). The study was conducted in accordance with the declaration of Helsinki (1964). Written informed consent was obtained from each patient or their next of kin.

### Participating ICUs and study population

We sent invitations to the 33 members of the Neuro-Critical Care Committee affiliated with the Chinese Association of Critical Care Physicians [21] by email, of which 24 agreed to participate in the study. All participating ICUs, including 14 general ICUs, 5 neuroscience ICUs, 3 surgical ICUs, and 2 emergency ICUs, are operated by the “closed” model, i.e. there is always an ICU physician presented in the ICU 24 h a day, 7 days a week [21, 22].

All adult patients admitted to the participating ICUs during the on-site investigation were enrolled in the present study. The exclusion criteria included age under 18 years, less than 24 h of ICU stay before the screening, and taking part in other studies.

The patients were predefined as belonging to the brain-injured group when their primary diagnoses were traumatic brain injury, stroke (subdivided into ischemic stroke, spontaneous intracerebral hemorrhage and subarachnoid hemorrhage), hypoxic-ischemic encephalopathy, elective craniotomy for brain tumor, intracranial infection, idiopathic epilepsy, and cranial venous sinus thrombosis [23]. Otherwise, the patients were classified as belonging to the non-brain-injured group.

### Data collection

A uniform case report form was designed to collect the data (Additional file 1). Data collection training was conducted for one researcher in charge of each participating ICU.

After enrolment, the hospital records were reviewed, and the following data were documented: demographics, history, diagnosis, length of ICU stay before enrolment, and the Glasgow Coma Scale (GCS) and Acute Physiology and Chronic Health Evaluation II scores at admission to the ICU. Nursing records during the 24-h period prior to enrolment were reviewed, and data were collected, including sequential organ failure assessment (SOFA) score, the presence of artificial airways (including oral or nasal endotracheal intubation or tracheostomy), the use of mechanical ventilation (invasive or non-invasive, modes and settings), the presence of arterial lines and central venous catheters, the presence of any types of drainage tubes (intracranial, lumbar, thoracic and intraperitoneal), the use of intracranial pressure monitoring, the performance of body temperature control (physical cooling for hyperthermia or hypothermia therapy), the presence of physical restraints, the PAD assessment (whether or not; if yes, the tools used), the use of analgesics, sedatives, anti-delirium drugs and neuromuscular blocking agents (whether or not; if yes, the name, the route and the drugs administered). The total daily dose of opioids was converted to the equianalgesic dose of fentanyl as previously reported [24].

Previous national survey of physicians showed the prevalence of PAD assessment tools used in Chinese ICUs [7]. The most common pain scores included the Visual Analogue Scale (VAS), Critical-Care Pain Observation Tool (CPOT), and Numerical Rating Scale (NRS). The Richmond Agitation-Sedation Scale (RASS) and Ramsay scale were the most popular scores for agitation-sedation assessment. Most of the physicians used the Confusion Assessment Method for the ICU (CAM-ICU) for delirium assessment. According to the recommendations in clinical guidelines [1, 3], we modified our case report form by adding items of Faces Pain Scale (FPS), Sedation Agitation Scale (SAS), and Intensive Care Delirium Screening Checklist (ICDSC) as the selection of assessment tool for pain, agitation-sedation, and delirium, respectively. An open option remained for each type of assessment. The development and implementation of PAD assessments require close collaboration of physicians and nurses [25]. This is also the case in China [26].

The patients were followed for 60 days or until discharge or death, whichever occurred first. The ICU and hospital records were reviewed, and the following data were collected: accidental removal of the catheter during the ICU stay, duration of mechanical ventilation, healthcare-associated infections, sepsis and septic shock during the ICU stay, the ICU length of stay (LOS), the hospital LOS, and in-hospital mortality. Hospital costs were also documented.

### On-site questionnaire survey

The on-site questionnaire survey was conducted in the same ICUs where the one-day point prevalence investigation was performed. The first draft of questionnaire was designed according to the clinical guidelines [1, 3] and previous survey studies in mainland China [7] and other countries [5, 6] related to PAD management. The final version (Additional file 2) was confirmed after a group discussion with experts including professors in critical care medicine, chief nurses, and professors in epidemiology and statistics.

We invited senior and junior physicians who were on-duty during the 24 h prior to the patients’ enrolment to complete the survey questionnaire on paper. It was documented if the physician refused to participate the survey.

### Study endpoints

We selected the primary endpoint as the prevalence of actual PAD assessment in our enrolled patients, which was compared with the attitudes of physicians reported in the questionnaire survey. Secondary endpoints included the rates of analgesic and sedative administration and clinical outcomes.

### Statistical analysis

We selected the primary endpoint as the prevalence of pain and agitation-sedation assessments using validated scales, which was reported approximately 40% critically ill patients by the European Critical Care Research Network [5]. Thus, a sample size of 369 is needed to achieve a precision of 95% confidence interval (CI) of the prevalence within 35 to 45%. The number of beds (*n* = 532) in recruited ICUs was enough to provide cases.

The prevalence and 95% CI of the actual practice and physicians’ perception of PAD management were calculated. Variables were compared between the brain-injured and non-brain-injured groups. Categorical variables are expressed as counts (percentages) and were compared by the chi-square test or Fisher exact test with small sample sizes. Continuous data are reported as medians with interquartile ranges and were compared using the unpaired Mann-Whitney U test.

All analyses were performed using the statistical software package SPSS (SPSS Inc., Chicago, IL, USA). Significance was indicated by *p* < 0.05.

## Results

### Recruited ICUs and patients

In the point prevalence study, we recruited 24 ICUs with 532 beds (21 [15–26] beds/ICU) in 20 hospitals (total beds: 37,047; 1550 [850–2727] beds/hospital) from six major administrative regions in China (Additional file 3: Fig. S1). Twelve hospitals were academically affiliated. Seventeen hospitals contributed data from one ICU only, two hospitals contributed data from two ICUs, and one hospital contributed data from three ICUs. The physician-to-bed ratio and nurse-to-bed ratios were 0.6 (0.4–0.7) and 2.3 (2.0–2.6), respectively.

**Fig. 1:**
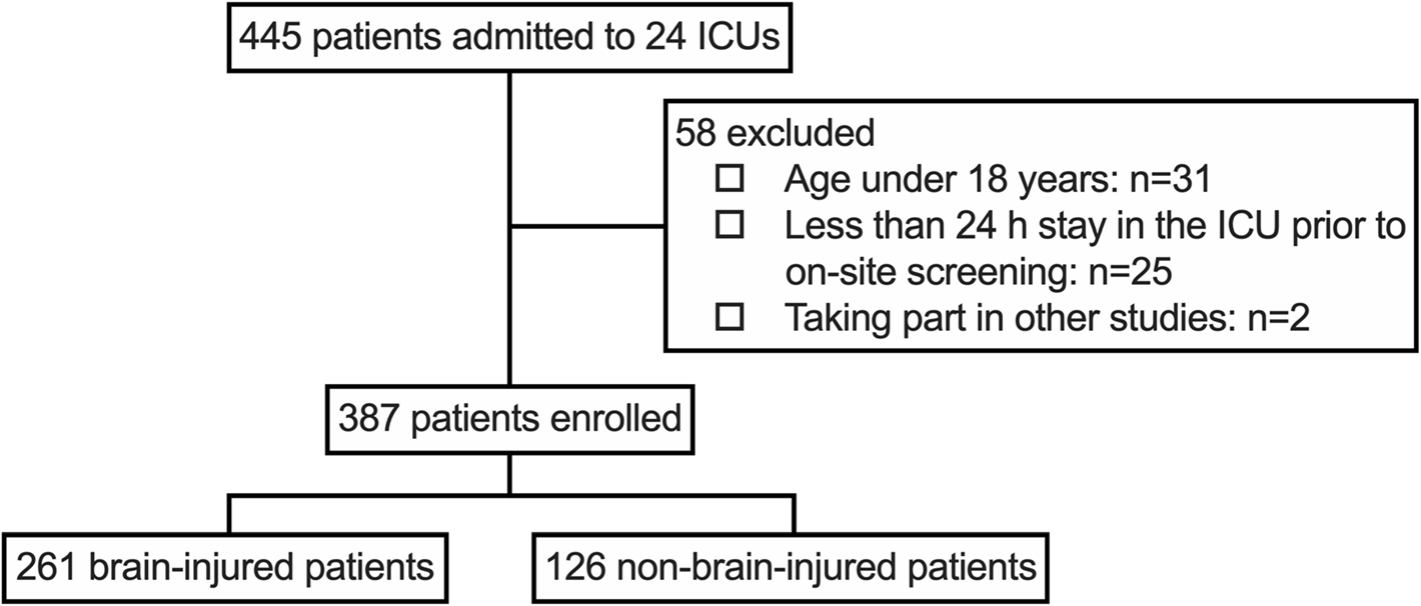
Patients flow chart

The investigation was started at 09:00 AM on January 8, 2019, and completed on March 9, 2019, after 60 days of follow-up. There were 445 patients in the ICUs during the on-site screening, of whom 58 were excluded because they were less than 18 years old (*n* = 31), had stayed in the ICU less than 24 h prior to the on-site screening (*n* = 25) or were taking part in other studies (*n* = 2). Finally, 387 patients were included in the study, with 261 (67.4%) brain-injured patients and 126 (32.6%) non-brain-injured patients (Fig. 1).

**Fig. 2:**
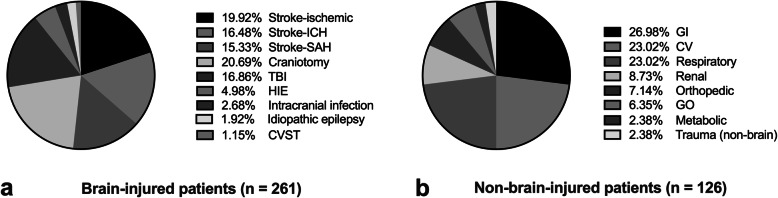
The primary diagnoses of enrolled patients

Figure 2 shows the main diagnosis. In patients with brain injury (*n* = 261), the most common types of brain injury were stroke (*n* = 135, 51.7%), elective craniotomy for brain tumors (*n* = 54, 20.7%), and traumatic brain injury (*n* = 44, 16.9%). In patients without brain injury (*n* = 126), the top three major diagnoses were gastrointestinal (*n* = 34, 27.0%), cardiovascular (*n* = 29, 23.0%) and respiratory system disease (=29, 23.0%).

Table 1 lists the characteristics of the patients. Compared with the non-brain-injured group, the brain-injured patients were younger (*p* < 0.001) and had less past medical history (*p* = 0.008), lower GCS at the ICU admission (*p* < 0.001), lower SOFA score during the 24 h prior to enrolment (*p* = 0.009), more artificial airways (*p* < 0.001) but less mechanical ventilation (*p* = 0.002), fewer arterial lines (*p* < 0.001) and drainage tubes (*p* = 0.006). Regarding outcome indicators, the incidence of sepsis and septic shock was significantly higher in non-brain-injured patients than in brain-injured patients (*p* < 0.001). No significant differences were found in LOS, mortality, and costs.

**Table 1:** Data collected from hospital and ICU nursing records for brain-injured and non-brain-injured patients

Patient Characteristics	All (*n* = 387)	Brain-injured (*n* = 261)	Non-brain-injured (*n* = 126)	*P*
Age, years	61 (47–76)	56 (44–72)	72 (54–79)	< 0.001
Male, n (%)	234 (60.5)	149 (57.1)	85 (67.5)	0.051
Any medical history, n (%)	224 (57.9)	139 (53.3)	85 (67.5)	0.008
Alcohol abuse, n (%)	9 (2.3)	3 (1.1)	6 (4.8)	0.064
History of smoke, n (%)	58 (15.0)	36 (13.8)	22 (17.5)	0.344
ICU LOS before enrolment, days	6 (1–14)	6 (1–14)	5 (1–13)	0.526
GCS at ICU admission	9 (5–14)	7 (4–11)	12 (9–15)	< 0.001
APACHE II at ICU admission	17 (11–22)	17 (10–22)	16 (11–22)	0.974
SOFA score on the day before study	5 (3–8)	5 (2–7)	6 (3–9)	0.009
Any artificial airway, n (%)	246 (63.6)	183 (70.1)	63 (50.0)	< 0.001
Oral intubation	121 (31.3)	79 (30.3)	42 (33.3)	0.542
Nasal intubation	20 (5.2)	17 (6.5)	3 (2.4)	0.093
Tracheostomy	105 (27.1)	87 (33.3)	18 (14.3)	< 0.001
Mechanical ventilation, n (%)				0.002
Invasive	187 (48.3)	125 (47.9)	62 (49.2)	
Non-invasive	11 (2.8)	2 (0.8)	9 (7.1)	
Presence of arterial line, n (%)	91 (23.5)	43 (16.5)	48 (38.1)	< 0.001
Presence of central venous catheter, n (%)	192 (49.6)	124 (47.5)	68 (54.0)	0.234
Presence of any drainage tubes, n (%)	101 (26.1)	57 (21.8)	44 (34.9)	0.006
Use of restraint, n (%)	233 (60.2)	165 (63.2)	68 (54.0)	0.081
Body temperature control, n (%)				0.184
Hyperthermia control	49 (12.7)	36 (13.8)	13 (10.3)	
Hypothermia therapy	5 (1.3)	5 (1.9)	0 (0)	
Outcomes				
Accidental removal of tubes, n (%)	8 (2.1)	6 (2.3)	2 (1.6)	0.645
All types of infection, n (%)	285 (73.6)	190 (72.8)	95 (75.4)	0.586
Sepsis, n (%)	65 (16.8)	45 (17.2)	20 (15.9)	
Septic shock, n (%)	23 (5.9)	4 (1.5)	19 (15.1)	
ICU mortality, n (%)	45 (11.6)	27 (10.3)	18 (14.3)	0.257
ICU LOS, days	15 (6–29)	16 (7–30)	14 (5–25)	0.218
Hospital mortality, n (%)	52 (13.4)	30 (11.5)	22 (17.5)	0.107
Hospital LOS, days	29 (17–58)	30 (18–58)	27 (16–56)	0.390
Hospital costs, CNY	132,000 (63,855–247,411)	132,000 (67,840–243,305)	131,579 (53,013–248,361)	0.540

*ICU* intensive care unit, *LOS* length of stay, *GCS* Glasgow Coma Scale, *APACHE* Acute Physiology and Chronic Health Evaluation, *SOFA* sequential organ failure assessment

Continuous data are shown as median (interquartile range)

### The actual practice of PAD management

**Fig. 3:**
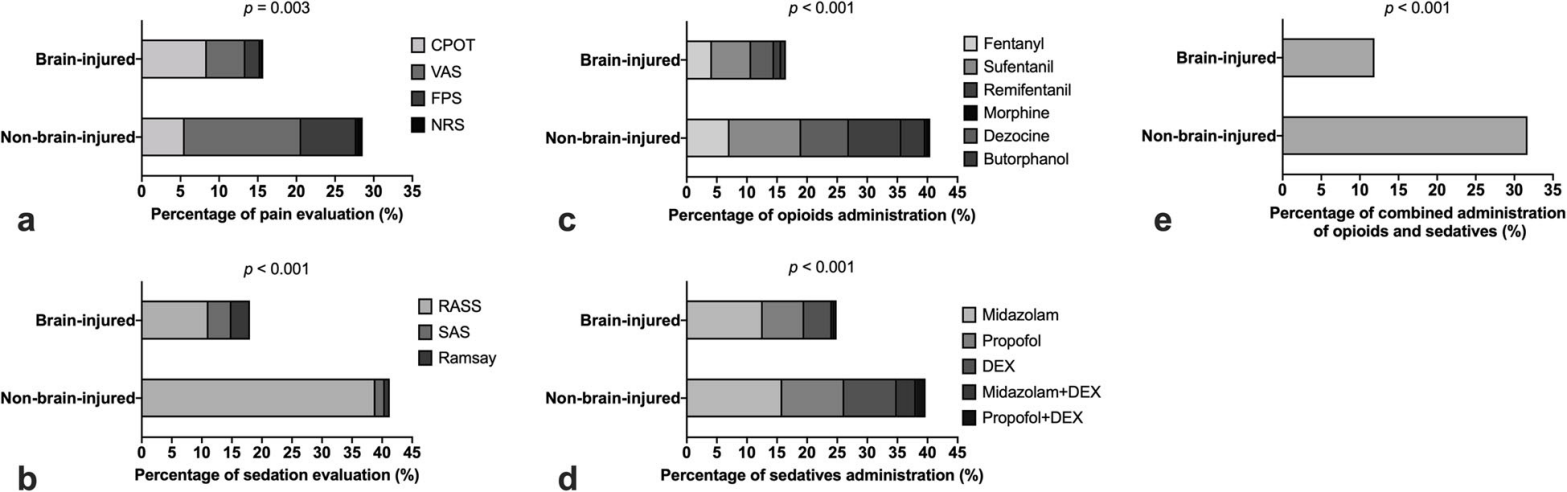
Prevalences of analgesia and agitation/sedation assessments and administrations. Data are shown as percentages. The prevalence of pain assessment using scale instruments were 19.9% (95% CI: 15.9–23.9%) (panel **a**). In patients receiving pain assessment (*n* = 77), four scales were used including VAS (*n* = 32, 41.6%), CPOT (*n* = 29, 37.7%), FPS (*n* = 14, 18.2%) and NRS (*n* = 2, 2.6%). The rate of pain assessment was significantly lower in brain-injured patients than non-brain-injured patients (*p* = 0.003). The prevalence of agitation/sedation assessment using scale instruments were 25.6% (95% CI: 21.2–29.9%) (panel **b**). In patients receiving agitation/sedation assessment (*n* = 99), three scales were used including RASS (*n* = 78, 78.8%), SAS (*n* = 12, 12.1%) and Ramsay scale (n = 9, 9.1%). The rate of agitation/sedation assessment was significantly lower in brain-injured patients than non-brain-injured patients (*p* < 0.001). The rate of administration of intravenous opioids was 24.3% (95% CI: 20.0–28.6%) (panel **c**). In patients receiving analgesics (*n* = 94), six opioids were administered including sufentanil (*n* = 32, 34.0%), fentanyl (*n* = 20, 21.3%), dezocine (*n* = 20, 21.3%), remifentanil (*n* = 14, 14.9%), butorphanol (*n* = 7, 7.4%) and morphine (*n* = 1, 1.1%). The use of opioids was less frequently in brain-injured patients than in non-brain-injured patients (*p* < 0.001). The rate of sedatives administration was 29.7% (95% CI: 25.1–34.3%) (panel **d**). In patients receiving sedatives (*n* = 115), midazolam, propofol, dexmedetomidine, midazolam combined with dexmedetomidine, and propofol combined with dexmedetomidine were used in 53 (46.1%), 31 (27.0%), 23 (20.0%), 5 (4.3%), and 3 (2.6%) patients, respectively. The use of sedatives was significantly less in brain-injured patients than in non-brain-injured patients (*p* < 0.001). The combination of opioids and sedatives was 18.3% (95% CI: 14.5–22.2%), which was administered less frequently in brain-injured patients than in non-brain-injured patients (*p* < 0.001, panel **e**)

**Fig. 4:**
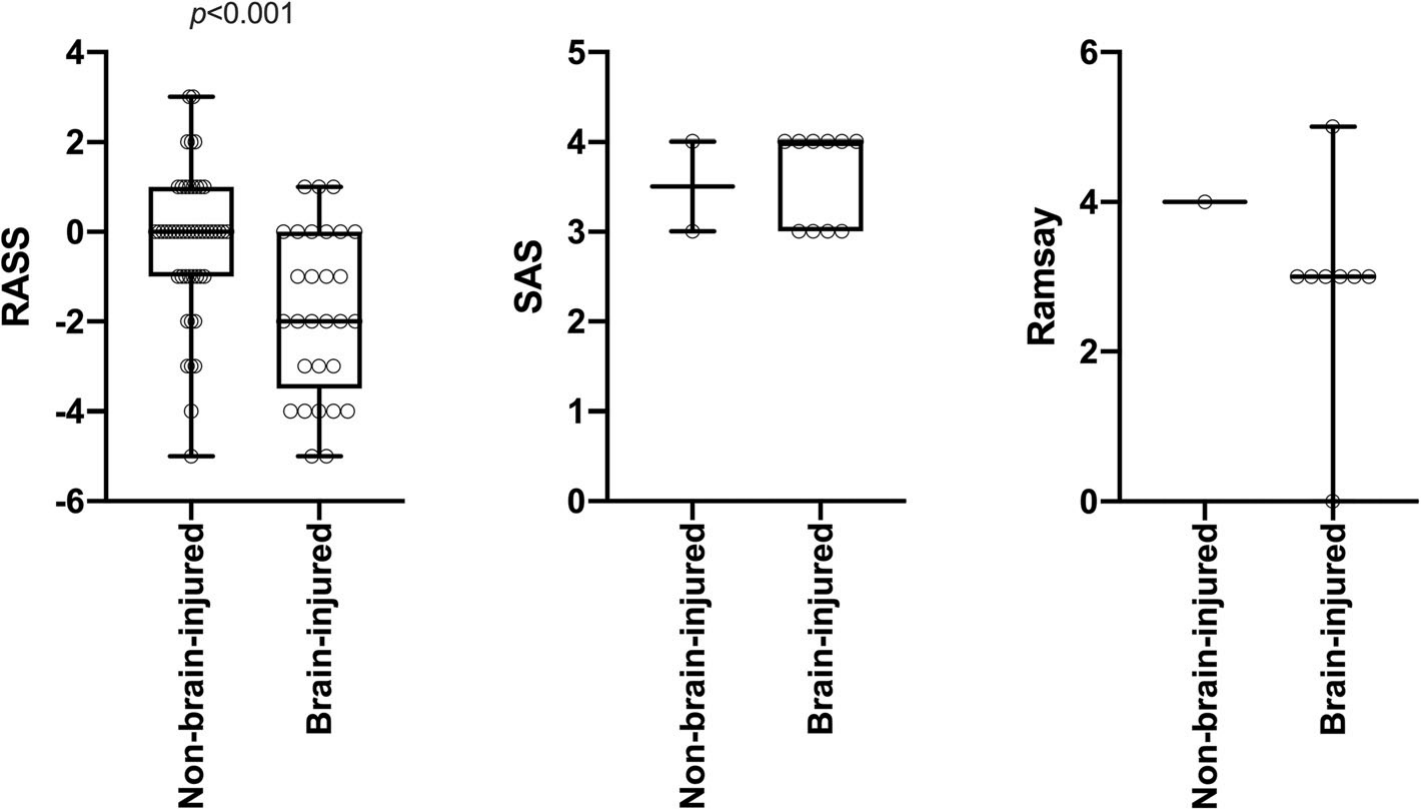
RASS, SAS and Ramsay scores in patients receiving agitation/sedation assessment. Data are shown as individual points with median, interquartile range and range. A total of 99 patients received agitation/sedation evaluation with 47 and 52 in the brain-injured and non-brain-injured groups, respectively. The RASS (*n* = 78, 78.8%) was the most frequently used tool. The RASS score was significantly higher in the non-brain-injured group (0 [− 1 − + 1]) than that in the brain-injured group (− 2 [− 4–0], *p* < 0.001)

The analgesia and sedation practices during the 24 h prior to enrolment are shown in Fig. 3. The prevalences of pain and agitation-sedation assessment using scale instruments were 19.9% (95% CI: 15.9–23.9%) and 25.6% (95% CI: 21.2–29.9%), respectively. The rates of the two types of assessments were significantly lower in brain-injured patients than non-brain-injured patients (Fig. 3a and b). Four tools were used for pain assessments, namely, the VAS, NRS, CPOT and FPS. Three tools were used for agitation-sedation assessments, namely, the RASS, SAS and Ramsay scale. Among the 99 patients receiving agitation-sedation evaluation (47 and 52 in the brain-injured and non-brain-injured groups, respectively), RASS (*n* = 78, 78.8%) was the most frequently used tool. The RASS score was significantly higher in the non-brain-injured group (0 [− 1 − + 1]) than that in the brain-injured group (− 2 [− 4–0], *p* < 0.001) (Fig. 4).

**Fig. 5:**
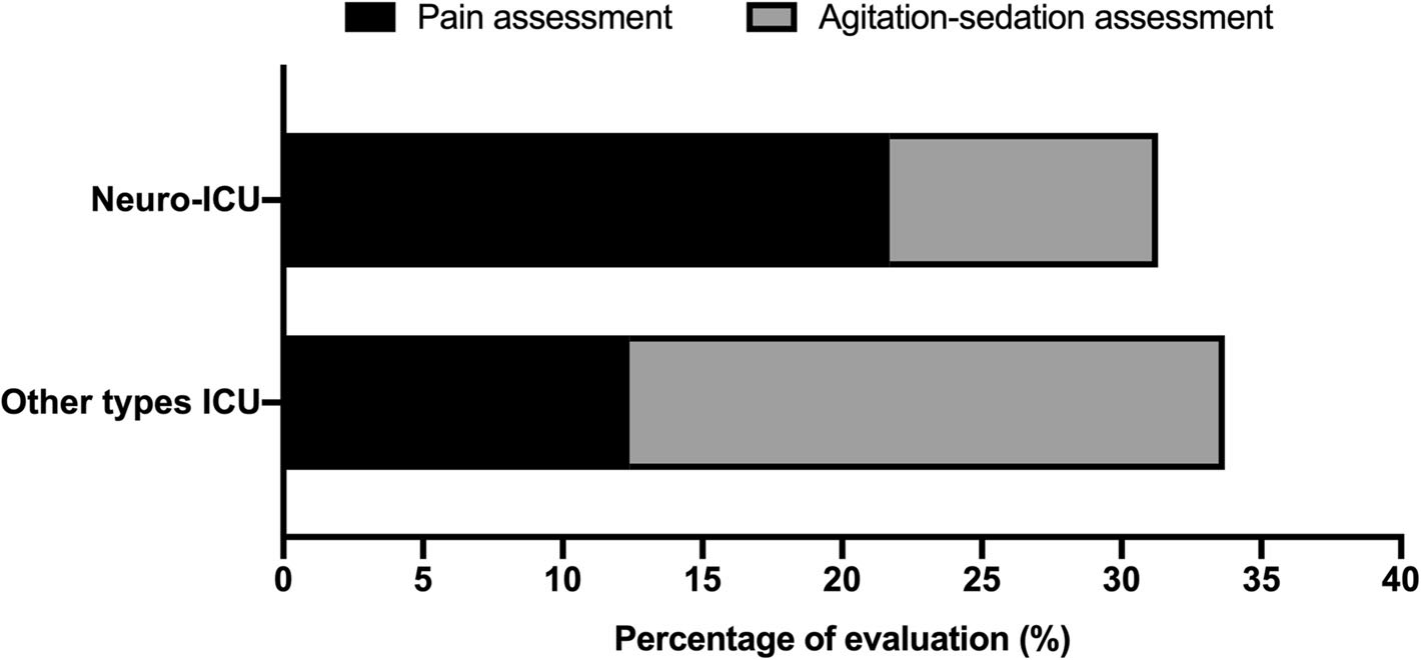
Rate of assessment of pain and agitation-sedation in brain-injured patients (*n* = 261) admitted to neuro-ICUs (*n* = 83) and other types of ICUs (*n* = 178). Compared to patients admitted to other types of ICUs, pain assessment was performed more often (21.7% vs. 12.4%, *p* = 0.05) but agitation-sedation assessment was performed less often (9.6% vs. 21.3%, *p* = 0.02) in patients admitted to neuro-ICUs

In 261 brain-injured patients, there were 83 (31.8%) and 178 (68.2%) admitted to neuro-ICUs and other types of ICUs, respectively. Although the overall rate of assessment of pain and agitation-sedation did not differ between patients admitted to neuro-ICUs and other types of ICUs (31.3% vs. 33.7%, *p* = 0.810), pain assessment was performed more often (21.7% vs. 12.4%, *p* = 0.05) but agitation-sedation assessment was performed less often (9.6% vs. 21.3%, *p* = 0.02) in patients admitted to neuro-ICUs compared to those admitted to other types of ICUs (Fig. 5).

The rates of administration of intravenous opioids, sedatives and the combination of the two types of agents were 24.3% (95% CI: 20.0–28.6%), 29.7% (95% CI: 25.1–34.3%) and 18.3% (95% CI: 14.5–22.2%), respectively. The three types of agents were administered less frequently in brain-injured patients than in non-brain-injured patients (Fig. 3c, d and e). The most commonly used opioids were fentanyl, sufentanil and dezocine. Remifentanil was also commonly used in non-brain-injured patients (Fig. 3c). The most commonly used sedatives were midazolam, propofol and dexmedetomidine (Fig. 3d).

Delirium assessment was only performed in three patients (0.8, 95% CI: 0.1–1.7%) using the CAM-ICU; the patients were two brain-injured patients and one non-brain-injured patient. Anti-delirium agents were used in six patients (three in the brain-injured group and three in the non-brain-injured group), with four administered haloperidol and two administered olanzapine. No patient received neuromuscular blocking agents during the 24 h prior to enrolment.

**Fig. 6:**
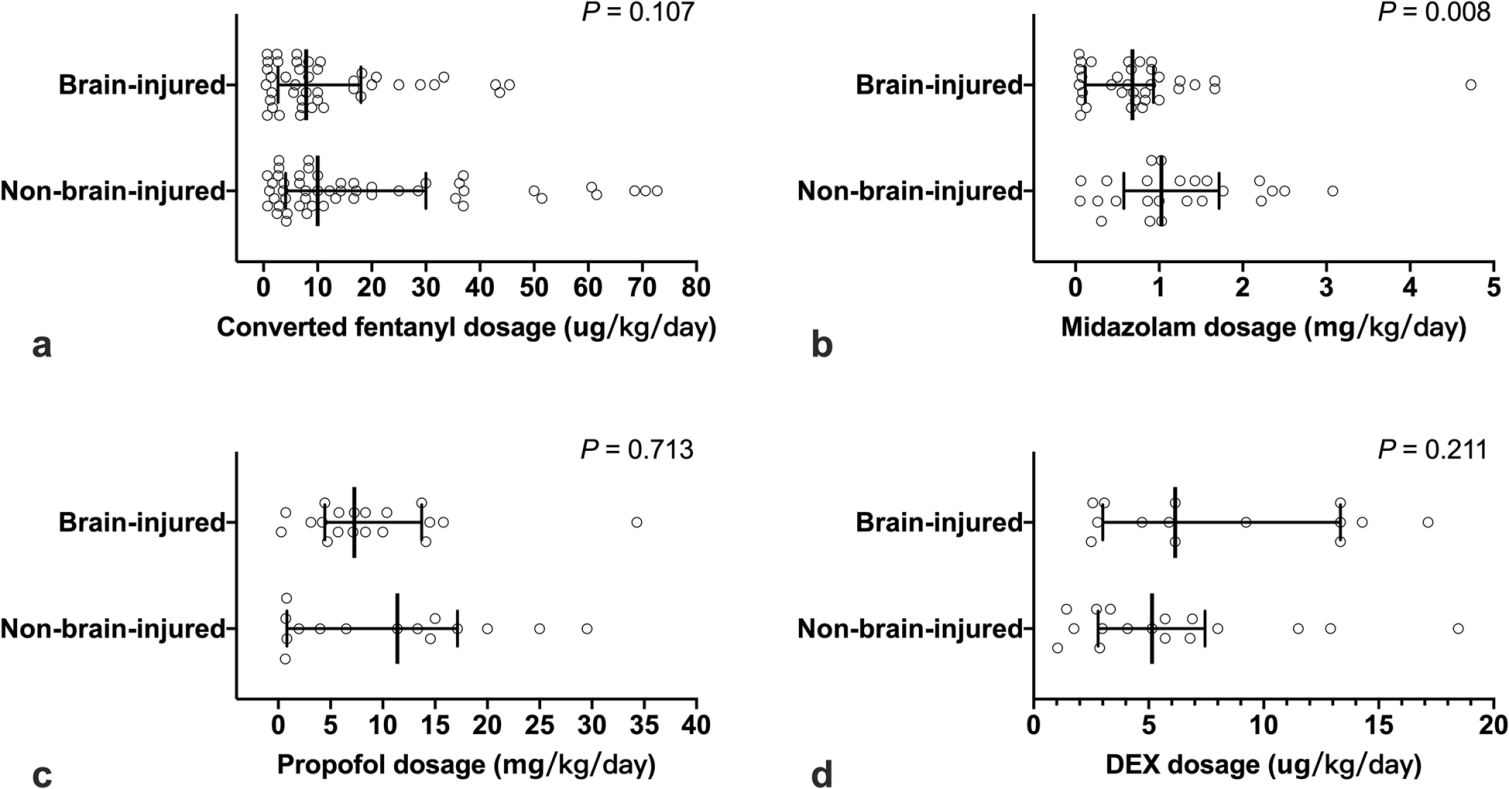
Cumulative doses of opioids and sedatives used during 24 h prior to on-site investigation in brain-injured and non-brain-injured patients. Data are presented as individual values and median with interquartile range

In patients receiving opioids and/or sedatives, a higher dose of midazolam was found in non-brain-injured patients (*n* = 61) than in brain-injured patients (*n* = 77), but no significant differences in the doses of other sedatives and opioids were found between the two groups (Fig. 6).

### Physicians’ replies to the questionnaire survey

During the on-site questionnaire survey, no physician refused to participate. Among the 24 participating ICUs, questionnaire surveys were collected from 91 physicians (3 [2–5]/unit), of whom 42 (46.2%) and 49 (53.8%) were senior and junior physicians, respectively. Analyses of the surveys are shown in Additional file 4.

Among the 91 physicians taking part in the survey, 63 (69.2, 95% CI: 59.6–78.9%) reported that there was a written analgesia and sedation protocol in their units. There were 64 (70.3, 95% CI: 60.8–79.9%) and 75 (82.4, 95% CI: 74.4–90.4%) physicians who reported the routine use of pain and agitation-sedation scale assessment, respectively (Table 2). The three most frequently used pain scale instruments were the VAS, NRS and CPOT. Three sedation scales were reported, namely, the RASS, SAS and Ramsay scale. Forty-eight physicians (52.7, 95% CI: 42.3–63.2%) reported daily screening for delirium using the CAM-ICU or ICDSC.

**Table 2:** The routine use of pain, agitation and delirium scale assessment: results of questionnaire survey collected from 91 physicians

Items	Number (%)
Routinely use a scale/score for pain assessment	64 (70.3%)
Visual Analogue Scale	24 (37.5%)
Numerical Rating Scale	12 (18.8%)
Verbal Rating Scale	9 (14.1%)
Faces Pain Scale	5 (7.8%)
Behavioral Pain Scale	3 (4.7%)
Critical-care Pain Observation Tool	11 (17.2%)
Others	0
Routinely use a scale/score for agitation/sedation assessment	75 (82.4%)
Richmond Agitation-Sedation Scale	55 (73.3%)
Sedation Agitation Scale	13 (17.3%)
Ramsay scale	7 (9.3%)
Motor Activity Assessment Scale	0
Others	0
Routinely screen patients daily for delirium	48 (52.7%)
Confusion Assessment Method for the ICU	43 (89.6%)
Intensive Care Delirium Screening Checklist	5 (10.4%)
Others	0

*ICU* intensive care unit

The first-choice opioids were fentanyl, sufentanil and remifentanil. The first-choice sedatives were midazolam, dexmedetomidine and propofol. Forty-eight (52.7, 95% CI: 42.3–63.2%) physicians reported very frequent/frequent combined use of analgesia and sedation.

## Discussion

We found that the point prevalence of PAD assessment was suboptimal, especially for delirium screening, in Chinese ICUs. A significant gap existed between the actual practice and the physicians’ perception of the practice. To the best of our knowledge, this is the first study reporting the real practice of PAD management in Chinese ICUs.

In accordance with the results of previous studies [5, 6], we also found a perceived and actual practice gap in the clinical performance of PAD assessment. More than half of the physicians reported the routine use of PAD scale assessments during the on-site questionnaire survey, whereas the assessment of pain and agitation-sedation was only performed in approximately 20 to 25% of patients which was lower than previous reports (43 to 88%) [5, 6]. Surprisingly, the actual delirium screening rate was extremely low (less than 1%) in our group of patients. This was in contrast to the results from an international point prevalence study, in which the rate of delirium assessment was reported as 48% with the use of a valid score of 27% [5]. The nurse-to-bed ratio and workload might be related to the lower rate of pain and agitation-sedation assessment, but could not explain the situation of delirium assessment. We speculated that the reasons for the low rate of delirium assessment might be multifaceted, such as continuing medical education, guideline implementation, and communication between physicians and nurses. However, these hypotheses need further confirmation.

Early quality improvement studies have shown that the routine incorporation of pain and agitation-sedation assessments into clinical practice can reduce the incidence of pain and agitation, reduce the duration of mechanical ventilation and rate of nosocomial infections, and decrease the need for analgesics and sedatives [27, 28]. Recent studies have also shown that implementing a guideline-derived comprehensive bundle can improve overall outcomes in critically ill patients [29, 30]. In the present study, we performed a chart review of the nursing records and conducted an on-site physician questionnaire survey in the same ICUs. All invited physicians completed the survey questionnaire, which included some simple questions focused on the PAD assessments (Additional file 2). These methods are facilitated to reveal the gap between performance and perception. Although a prospective cohort study showed that PAD management was significantly improved after the publication of guidelines by the Society of Critical Care Medicine, actual practice varied widely across international regions [31]. Our results highlight the need for a quality improvement program for PAD management in Chinese ICUs. This program should comprise promotion of current PAD guidelines, the establishment of PAD assessment routine, encouragement of collaboration among ICU medical personnel especially for physicians and nurses, and monitoring patient’s outcome.

Although evidence has shown that pain and sedation assessments are feasible and reliable in the majority of brain-injured patients [12, 13], barriers to the routine application may also exist due to physician perception of consciousness impairment in this population [17–20]. A previous study demonstrated that different monitoring and treatment protocols were employed in neurological and non-neurological patients admitted to ICUs [32]. Our results showed that, compared to non-brain-injured patients, ICU-admitted brain-injured patients received fewer pain and agitation-sedation assessments, with a rate of performance as low as 16 to 18%. Our results suggest that future studies are warranted to optimize pain and agitation-sedation management in critically brain-injured patients.

Diagnosis of delirium in brain-injured patients with coma is controversial. According to the Diagnostic and Statistical Manual of Mental Disorders, 5th edition [33], the disturbances in attention and cognition are not explained by another preexisting, established, or evolving neurocognitive disorder and do not occur in the context of a severely reduced level of arousal, such as coma. However, recent evidence has also shown that delirium is prevalent in critically ill neurological patients and might be associated with unfavorable clinical outcomes [34]. Assessment tools used in the general ICUs, such as the CAM-ICU and ICDSC, are also applicable in patients with brain injury [12, 13]. Current consensus statements recommend that delirium should be routinely monitored and managed in critically ill neurological patients [14, 15]. Our results indicate the necessity of establishing delirium monitoring routine in this population.

In our patients without brain injury, the rates of administration of opioids (40.8%), sedatives (41.8%) and the combination of the two types of agents (31.7%) were comparable to those reported by Richards-Belle et al. in the United Kingdom (41.5, 44.6 and 32.7% for analgesics, sedatives and the combination of the two, respectively) [6]. The most commonly used opioids in the present study were sufentanil and fentanyl, which were similar to those in previous reports [5, 6]. However, the most frequently used sedative was midazolam in our patients, which is different from the current sedation protocol with the dominant use of propofol and dexmedetomidine [1–3]. Our results showed that opioids and sedatives were less common in brain-injured patients than in non-brain-injured patients. Clinical guidelines also recommended controlling pain before sedation [1–3]. However, approximately 10% of patients (the difference in the use of sedatives and the combined use of sedatives and opioids) were administered sedatives without analgesics, indicating another potential area of quality improvement in Chinese ICUs. The association of the choice of analgesics and sedatives with clinical outcomes in critically brain-injured patients needs further investigation.

There are limitations in the present study. First, the limitations inherent in point prevalence studies and questionnaire surveys could not be avoided in the present study. Because the questionnaires used in survey studies on PAD management were relatively confirmative [5–7], we did not perform the psychometric evaluation of our self-developed questionnaire. Additionally, only 24 ICUs with 532 beds from ten provinces in China were recruited. Due to the relatively small number of cases, we did not analyze the specific sedation in different diseases. However, in this study, patients were enrolled and physicians were recruited from the same ICUs, providing the opportunity to investigate the gap between perceived and actual clinical PAD management practices. Our results highlighted the importance of quality improvement in this area. Second, the PAD assessments are usually performed by the nurses. We did not conduct a questionnaire survey in nurses. Additionally, we did not perform the chart review of the physician’s notes because PAD assessments are routinely documented in the nursing records in Chinese ICUs. However, the actual rate of PAD assessments reflects the real-world situation. The development and implementation of PAD assessment protocol require the cooperation of physicians and nurses. Therefore, our data also reflect the gap between perception and performance. Third, because the main propose in the present study was to investigate the gap between the perception and actual practice of physicians in PAD assessment, we did not collect all the items recommended in the PAD guidelines [3], such as the daily interruption of sedation and non-pharmacological interventions for pain and delirium management. We will continue to collect such data and implement further quality improvement projects in future work. Finally, we could not confirm the purpose of analgesia and sedation from ICU nursing records. For critically brain-injured patients, analgesia and sedation are also used to control intracranial pressure, facilitate therapeutic hypothermia and maintain the balance between cerebral oxygen demand and consumption [9, 10]. Only 8 and 5 patients were receiving intracranial pressure monitoring and therapeutic hypothermia in the brain-injured group. Thus, the specific administration of analgesia and sedation for cerebral protection would seldom have occurred in the brain-injured patients enrolled in the present study.

## Conclusions

In conclusion, in critically ill patients admitted to the Chinese ICUs, we found that the actual PAD assessment rate was suboptimal, especially with regard to the delirium screening. A gap existed between physician perception and actual practice in clinical performance. Our results highlight the need for prompt quality improvement and the optimization of practices of PAD management in ICUs in China. A standard PAD management protocol should be established for critically brain-injured patients.

## Supplementary Information

**Additional file 1.** Case report form for cross-sectional investigation.

**Additional file 2.** Predefined survey questionnaire for on-site survey.

**Additional file 3: Figure S1**. Distribution of 20 recruited hospitals.

**Additional file 4.** Analyses of the questionnaire surveys.

## Data Availability

The datasets analyzed during the current study are available from the corresponding author on reasonable request.

## Abbreviations

ICU: intensive care unit
PAD: pain, agitation-sedation and delirium
GCS: Glasgow Coma Scale
SOFA: sequential organ failure assessment
LOS: length of stay
CI: confidence interval
ICH: spontaneous intracerebral hemorrhage
SAH: subarachnoid hemorrhage
HIE: hypoxic-ischemic encephalopathy
CVST: cranial venous sinus thrombosis
GI: gastrointestinal
CV: cardiovascular
GO: gynecological and obstetrical
VAS: Visual Analogue Scale
NRS: Numerical Rating Scale
CPOT: Critical-Care Pain Observation Tool
FPS: Faces Pain Scale
RASS: Richmond Agitation-Sedation Scale
SAS: Sedation Agitation Scale
DEX: dexmedetomidine
CAM-ICU: the Confusion Assessment Method for the ICU
ICDSC: Intensive Care Delirium Screening Checklist
