# Supplementary material for: A gap existed between physicians’ perceptions and performance of pain, agitation-sedation and delirium assessments in Chinese intensive care units
Source: BMC Anesthesiol. 2021 Feb 25;21:61. doi: 10.1186/s12871-021-01286-w (PMC7905610; doi:10.1186/s12871-021-01286-w)
Supplement: Supplementary file 1 — Additional file 1. Case report form for cross-sectional investigation. [file 12871_2021_1286_MOESM1_ESM.pdf]

A gap existed between physicians' perceptions and performance of pain, agitation-sedation and delirium assessments in Chinese intensive care units

Kai Chen, Yan-Lin Yang, Hong-Liang Li, Dan Xiao, Yang Wang, Linlin Zhang, Jian-Xin Zhou

Additional file 1:

Case report form for cross-sectional investigation

Sedation practice in brain-injured patients in the intensive care units in China: a point prevalence study and a questionnaire survey

Case Report Form

Investigation site	□□□□□□□□□□□□
Investigator	□□□□
Screen No.	□□□□
Recruitment No.	□□□
Medical record No.	□□□□□□
Abbreviation name	□□□□
Date	201□-□□-□□

Illustrations

- This is a multicenter one-day point prevalence study combined with questionnaire survey.
- The protocol was approved by the Institutional Review Board of Beijing Tiantan Hospital, Capital Medical University. Written informed consent was obtained from each patient or their next of kin. During the study period, no attempt was made to alter or affect the routine clinical practice of each participating ICU.
- All recorded data must be timely, accurate and complete.
- Every page and every item of the CRF must be completed.
- Fill "√" in the "□" to indicate selection. "NA" to indicate "cannot provide" or "not applicable".
- Patient's abbreviated name should be recorded in the form of left aligned acronym of Chinese phonetic alphabet. For example: Zhang Wei should be recorded as Z W, and Zhang Xiao-Wei should be recorded as Z X W.
- Numeric data should be recorded with decimals provided as □.□ in the form.
- If error(s) occurs in filling data, please use ONE strikethrough line and refill the correct data, and sign name of the corrector and date of correction.
  - Do not cover the original data.
  - Do not use the eraser or correction fluid.
  - Do not cross more than one strikethrough line.
- If investigators have any questions during the study, please contact Drs. Jian-Xin Zhou, Linlin Zhang:
  - **Dr. Jian-Xin Zhou: 010 59975098**
  - **Dr. Linlin Zhang: 010 59978451**

- Inclusion criterion: All adult patients admitted to the participating ICUs during the on-site screening, regardless of the primary diagnosis.
- Exclusion criteria: please check the hospital records and examine the patient for exclusion:
    - Age < 18-years-old ☐ Yes ☐No
    - Less than 24 hours of ICU stay before screening ☐ Yes ☐No
    - Taking part in other studies ☐ Yes ☐No
    - Refusing to participate ☐ Yes ☐No

If there is no exclusion criterion, please go next page.

Investigator recruitment ☐☐☐☐

Data collection from hospital records

Sex	☐ male ☐ female	
Age	☐☐ years	
Height	☐☐☐ cm	
Weight	☐☐☐ kg	
Date of admission to the hospital	201☐/☐☐MM/☐☐DD	
Date of admission to the ICU	201☐/☐☐MM/☐☐DD	
Primary diagnosis		
Brain injuries	☐ no ☐ yes, if yes, please tick one box below	
Types of brain injuries	Traumatic brain injury	☐yes ☐no
	Brain tumors	☐yes ☐no
	Ischemic stroke	☐yes ☐no
	Spontaneous intracerebral hemorrhage	☐yes ☐no
	Subarachnoid hemorrhage	☐yes ☐no
	Idiopathic epilepsy	☐yes ☐no
	Intracranial infection	☐yes ☐no
	Hypoxic-ischemic encephalopathy	☐yes ☐no
	Others, please indicate___________________________	
Medical history	Hypertension	☐yes ☐no
	Coronary artery disease	☐yes ☐no
	Diabetes	☐yes ☐no
	Chronic obstructive pulmonary diseases	☐yes ☐no
	Chronic kidney diseases	☐yes ☐no
	Ischemic stroke	☐yes ☐no
	Alcohol abuse	☐yes ☐no
	History of smoke	☐yes ☐no
	Others, please indicate_________________________	
GCS at the ICU admission	☐☐ points: E (eyes) ☐; V (verbal) ☐; M (motor) ☐	
APACHE II at the ICU admission	☐☐ points	

Data collection from nursing records during the 24 hours prior to enrollment

Status

SOFA within 24 hours prior to enrollment	☐☐ points
Artificial airways	☐ oral ☐ nasal ☐ tracheostomy ☐others, please indicate:______
Mechanical ventilation	☐ no ☐ invasive ☐ non-invasive<br>if yes, please tick one box below
	Date of start: 201☐/☐☐MM/☐☐DD
	☐PCV ☐P-A/C ☐VCV ☐V-A/C ☐IPPV ☐PRVC ☐BIPAP<br>☐APRV ☐P-SIMV ☐V-SIMV ☐PSV ☐CPAP ☐ others,<br>please indicate:________________________
	PEEP: ☐☐cmH_2O; FiO_2: ☐☐% VT: ☐☐☐ml
Arterial lines	☐ yes ☐ no
Central venous catheters	☐ yes ☐ no
ICP monitor	☐ no ☐ yes, please record: ☐☐mmHg
Any types of drainage tubes and colostomy	☐ventricle ☐lumbar ☐epidural ☐subdural ☐thoracic<br>☐intraperitoneal ☐pelvic ☐mediastinal ☐gastrostomy<br>☐intestinal colostomy ☐bladder colostomy<br>☐others, please indicate:_______________
Physical restraints	☐ yes ☐ no
Body temperature control	☐ no ☐ physical cooling for hyperthermia<br>☐ hypothermia therapy

Data collection from nursing records during the 24 hours prior to on-site investigation

Assessments

Pain assessment	☐ no ☐ yes, please indicate		
	☐ CPOT	☐☐ points	☐☐/d
	☐ VAS	☐☐ points	☐☐/d
	☐ FPS	☐☐ points	☐☐/d
	☐ NRS	☐☐ points	☐☐/d
	☐ others, please indicate:_______________	☐☐ points	☐☐/d
Agitation and sedation assessments	☐ no ☐ yes, please indicate		
	☐ RASS	☐☐ points	☐☐/d
	☐ SAS	☐☐ points	☐☐/d
	☐ Ramsay	☐☐ points	☐☐/d
	☐ others, please indicate:_______________	☐☐ points	☐☐/d
Delirium assessment	☐ no ☐ yes, please indicate		
	☐ CAM-ICU	☐ yes ☐ no	☐☐/d
	☐ ICDSC	☐ yes ☐ no	☐☐/d
	☐ others, please indicate:_______________	☐ yes ☐ no	☐☐/d

Data collection from nursing records during the 24 hours prior to on-site investigation

Medications

Analgesics	☐ no ☐ yes, please indicate		
	☐ Fentanyl	☐ IV ☐ IM ☐ PO	☐☐☐☐μg/d
	☐ Sufentanil	☐ IV ☐ IM ☐ PO	☐☐☐☐μg/d
	☐ Remifentanil	☐ IV ☐ IM ☐ PO	☐☐☐☐μg/d
	☐ Morphine	☐ IV ☐ IM ☐ PO	☐☐mg/d
	☐ Pethidine	☐ IV ☐ IM ☐ PO	☐☐mg/d
	☐ Tramadol	☐ IV ☐ IM ☐ PO	☐☐mg/d
	☐ Dezocine	☐ IV ☐ IM ☐ PO	☐☐mg/d
	☐ Butorphanol	☐ IV ☐ IM ☐ PO	☐☐mg/d
	☐ Flupiprofen ester	☐ IV ☐ IM ☐ PO	☐☐mg/d
	☐ Paracetamol oxycodone	☐ IV ☐ IM ☐ PO	☐☐mg/d
	☐ Acetaminophen	☐ IV ☐ IM ☐ PO	☐☐mg/d
	☐ others, please indicate:_______________	☐ IV ☐ IM ☐ PO	☐☐mg/d
	☐ others, please indicate:_______________	☐ IV ☐ IM ☐ PO	☐☐mg/d
Sedatives	☐ no ☐ yes, please indicate		
	☐ Midazolam	☐ IV ☐ IM ☐ PO	☐☐mg/d
	☐ Propofol	☐ IV ☐ IM ☐ PO	☐☐mg/d
	☐ Dexmedetomidine	☐ IV ☐ IM ☐ PO	☐☐μg/d
	☐ Diazepam	☐ IV ☐ IM ☐ PO	☐☐mg/d
	☐ Lorazepam	☐ IV ☐ IM ☐ PO	☐☐mg/d
	☐ Estazolam	☐ IV ☐ IM ☐ PO	☐☐mg/d
	☐ others, please indicate:_______________	☐ IV ☐ IM ☐ PO	☐☐mg/d
	☐ others, please indicate:_______________	☐ IV ☐ IM ☐ PO	☐☐mg/d
Muscle relaxants	☐ no ☐ yes, please indicate		
	☐ Rocuronium	IV	☐☐mg/d
	☐ Vecuronium	IV	☐☐mg/d
	☐ Pancuronium	IV	☐☐mg/d
	☐ Cis-atracurium	IV	☐☐mg/d
	☐ others, please indicate:_______________	IV	☐☐mg/d
Anti-delirium	☐ no ☐ yes, please indicate		
	☐ Haloperidol	☐ IV ☐ IM ☐ PO	☐☐mg/d
	☐ Chlorpromazine	☐ IV ☐ IM ☐ PO	☐☐mg/d
	☐ Promethazine	☐ IV ☐ IM ☐ PO	☐☐mg/d
	☐ Olanzapine	☐ IV ☐ IM ☐ PO	☐☐mg/d
	☐ Risperidone	☐ IV ☐ IM ☐ PO	☐☐mg/d
	☐ others, please indicate:_______________	☐ IV ☐ IM ☐ PO	☐☐mg/d

Outcome measures

Type of follow-up (please fill "√" one)

☐ Discharge from the hospital

☐ Death in the ICU

☐ Death in the hospital

☐ At 60 days after enrollment

Please review the ICU and hospital records

During ICU stay after enrollment	Accidental removal of the catheter	☐no ☐yes, please indicate: ☐☐times
	Duration of mechanical ventilation	☐☐☐days
	Hospital acquired infections	☐Pneumonia ☐Urinary tract infection<br>☐Intracranial infection ☐Surgical site infection<br>☐Bacteremia ☐CRBSI<br>☐others, please indicate:___________________
	Sepsis	☐no ☐Sepsis ☐Septic shock
	Survival when discharge from ICU	☐yes ☐no ☐have not discharge yet
	Date of discharge from ICU	If yes, please indicate :201☐/☐☐MM/☐☐DD
	ICU length of stay (LOS)	☐☐☐☐days
During hospital stay after enrollment	Survival when discharge from hospital	☐yes ☐no ☐have not discharge yet
	Date of discharge from hospital	If yes, please indicate: 201☐/☐☐MM/☐☐DD
	Hospital LOS	☐☐☐☐days
	Hospital costs	☐☐☐☐☐☐☐.☐☐RMB
	GOS	If brain-injured, please indicate: ☐points
