# Supplementary material for: A gap existed between physicians’ perceptions and performance of pain, agitation-sedation and delirium assessments in Chinese intensive care units
Source: BMC Anesthesiol. 2021 Feb 25;21:61. doi: 10.1186/s12871-021-01286-w (PMC7905610; doi:10.1186/s12871-021-01286-w)
Supplement: Supplementary file 2 — Additional file 2. Predefined survey questionnaire for on-site survey. [file 12871_2021_1286_MOESM2_ESM.pdf]

A gap existed between physicians' perceptions and performance of pain, agitation-sedation and delirium assessments in Chinese intensive care units

Kai Chen, Yan-Lin Yang, Hong-Liang Li, Dan Xiao, Yang Wang, Linlin Zhang, Jian-Xin Zhou

Additional file 2:

Predefined survey questionnaire for on-site survey

Questionnaire for analgesia and sedation practice

Hospital name:

____

ICU name:

____

Type of ICU (please tick one box):

General □; Surgical □; Medical □ Neuro □; other □ please indicate ___________________________

Name (person completing questionnaire)

____

Job title (please tick one box)

Senior □

- Lead/Director □
- Attending physician □

Junior □

- Resident □
- Research fellow □
- Trainee □

Other (please indicate)

____

Questionnaire survey

1. Do you have a written analgesia and sedation protocol? (please tick one box)

Yes ☐ No ☐

2. Do you routinely use a scale/score for **PAIN** assessment? (please tick one box)

Yes ☐ No ☐

If Yes, which pain scale/score is routinely used? (please tick one box)

Visual Analogue Scale (VAS) ☐

Numerical Rating Scale (NRS) ☐

Verbal Rating Scale (VDS) ☐

Faces Pain Scale (FPS) ☐

Behavioral Pain Scale ☐

Critical-care Pain Observation Tool (CPOT) ☐

Others ☐ please indicate ____________________

3. Do you routinely use a scale/score for **AGITATION/SEDATION** assessment? (please tick one box)

Yes ☐ No ☐

If Yes, which agitation/sedation scale/score is routinely used? (please tick one box)

Richmond Agitation-Sedation Scale (RASS) ☐

Sedation Agitation Scale (SAS) ☐

Rampsy scale ☐

Motor Activity Assessment Scale (MAAS) ☐

Others ☐ please indicate ____________________

4. Do you use daily sedation interruption for sedated patients? (please tick one box)

Never ☐ Rarely ☐ Occasionally ☐ Frequently ☐ Very frequently ☐

5. Do you screen patients daily for delirium? (please tick one box)

Yes ☐ No ☐

If Yes, which tool is routinely used? (please tick one box)

Confusion Assessment Method for the ICU (CAM-ICU) ☐

Intensive Care Delirium Screening Checklist (ICDSC) ☐

Others ☐ please indicate ______________

6. Please indicate the three most frequently used opioids (please mark 1, 2, 3)

Morphine: ☐

Fentanyl: ☐

Sufentanil: ☐

Remifentanil: ☐

Dezocine: ☐

Butorphanol: ☐

Others please indicate: ____________

7. Please indicate the three most frequently used opioids (please mark 1, 2, 3)

Propofol: ☐

Midazolam: ☐

Diazepam: ☐

Lorazepam: ☐

Dexmedetomidine: ☐

Others please indicate: ________________

8. Do you use combined analgesia and sedation for your patients? (please tick one box)

Never ☐ Rarely ☐ Occasionally ☐ Frequently ☐ Very frequently ☐

9. Do you use continuous infusion of neuromuscular blocking agent for sedated and ventilated patients? (please tick one box)

Never ☐ Rarely ☐ Occasionally ☐ Frequently ☐ Very frequently ☐

THANK YOU FOR YOUR TIME
