# Supplementary material for: A gap existed between physicians’ perceptions and performance of pain, agitation-sedation and delirium assessments in Chinese intensive care units
Source: BMC Anesthesiol. 2021 Feb 25;21:61. doi: 10.1186/s12871-021-01286-w (PMC7905610; doi:10.1186/s12871-021-01286-w)
Supplement: Supplementary file 3 — Additional file 3: Figure S1. Distribution of 20 recruited hospitals. [file 12871_2021_1286_MOESM3_ESM.pdf]

A gap existed between physicians' perceptions and performance of pain, agitation-sedation and delirium assessments in Chinese intensive care units

Kai Chen, Yan-Lin Yang, Hong-Liang Li, Dan Xiao, Yang Wang, Linlin Zhang, Jian-Xin Zhou

Additional file 3:

Figure S1. Distribution of 20 recruited hospitals.

Figure S1. Distribution of 20 recruited hospitals.

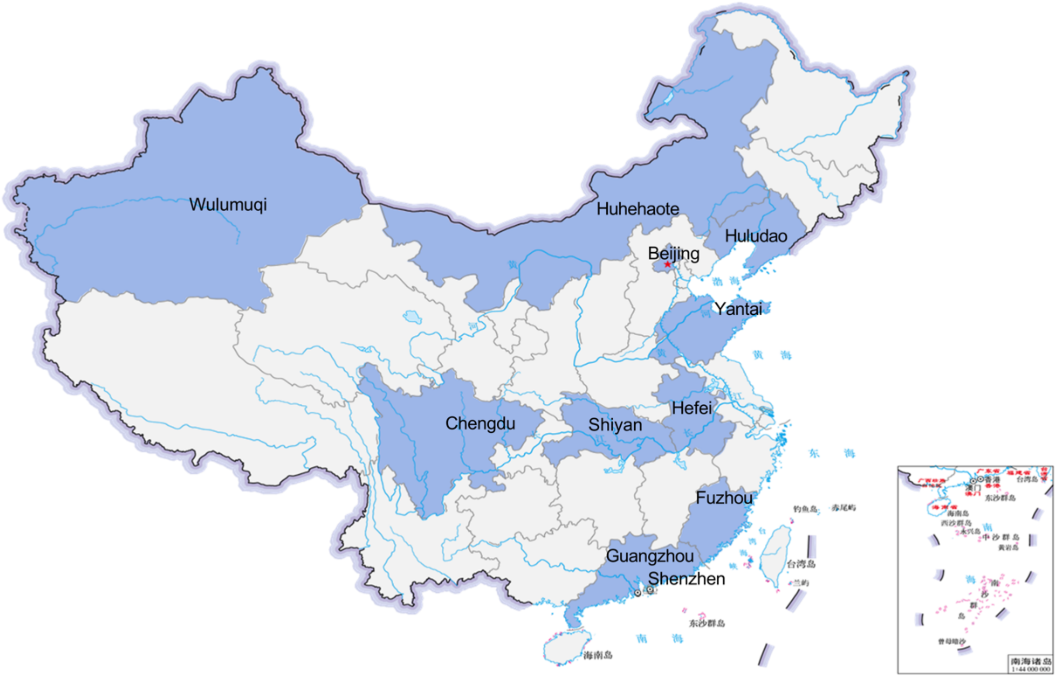

Twenty recruited hospitals are distributed to six Chinese administrative regions including WestNorth, North, EastNorth, East, South, and WestSouth.
