# Supplementary material for: A gap existed between physicians’ perceptions and performance of pain, agitation-sedation and delirium assessments in Chinese intensive care units
Source: BMC Anesthesiol. 2021 Feb 25;21:61. doi: 10.1186/s12871-021-01286-w (PMC7905610; doi:10.1186/s12871-021-01286-w)
Supplement: Supplementary file 4 — Additional file 4. Analyses of the questionnaire surveys. [file 12871_2021_1286_MOESM4_ESM.pdf]

A gap existed between physicians' perceptions and performance of pain, agitation-sedation and delirium assessments in Chinese intensive care units

Kai Chen, Yan-Lin Yang, Hong-Liang Li, Dan Xiao, Yang Wang, Linlin Zhang, Jian-Xin Zhou

Additional file 4:

Analyses of the questionnaire surveys

Analysis for questionnaire survey

1. Do you have a written analgesia and sedation protocol?

Yes 63 (69.2%) No 28 (30.8%)

2. Do you routinely use a scale/score for **PAIN** assessment?

Yes 64 (70.3%) No 27 (29.7%)

If Yes, which pain scale/score is routinely used? (please tick one box)

Visual Analogue Scale (VAS)	24 (37.5%)
Numerical Rating Scale (NRS)	12 (18.8%)
Verbal Rating Scale (VRS)	9 (14.1)
Faces Pain Scale (FPS)	5 (7.8%)
Behavioral Pain Scale	3 (4.7%)
Critical-care Pain Observation Tool (CPOT)	11 (17.2%)
Others	0

3. Do you routinely use a scale/score for **AGITATION/SEDATION** assessment?

Yes 75 (82.4%) No 16 (17.6%)

If Yes, which agitation/sedation scale/score is routinely used? (please tick one box)

Richmond Agitation-Sedation Scale (RASS)	55 (73.3%)
Sedation Agitation Scale (SAS)	13 (17.3%)
Rampsy scale	7 (9.3%)
Motor Activity Assessment Scale (MAAS)	0
Others	0

4. Do you use daily sedation interruption for sedated patients? (please tick one box)

Never	17 (18.7%)
Rarely	10 (11.0%)
Occasionally	11 (12.1%)
Frequently	22 (24.2%)
Very frequently	31 (34.1%)

5. Do you screen patients daily for delirium? (please tick one box)

Yes 48 (52.7%) No 43 (47.3%)

If Yes, which tool is routinely used? (please tick one box)

Confusion Assessment Method for the ICU (CAM-ICU)	43 (89.6%)
Intensive Care Delirium Screening Checklist (ICDSC)	5 (10.4%)
Others	0

6. Please indicate the three most frequently used opioids (please mark 1, 2, 3)

	First choice	Second choice	Third choice
Morphine:	6 (6.6%)	9 (9.9%)	39 (42.9%)
Fentanyl:	34 (37.4%)	32 (35.2%)	15 (16.5%)

Sufentanil:	27 (29.7%)	17 (18.7%)	11 (12.1%)
Remifentanil:	13 (14.3%)	31 (34.1%)	16 (17.6%)
Dezocine:	10 (11.0%)	1 (1.1%)	4 (4.4%)
Butorphanol:	1 (1.1%)	1 (1.1%)	6 (6.6%)

7. Please indicate the three most frequently used sedatives (please mark 1, 2, 3)

	First choice	Second choice	Third choice
Midazolam:	50 (54.9%)	26 (28.6%)	15 (16.5%)
Propofol:	18 (19.8%)	44 (48.4%)	27 (29.7%)
Dexmedetomidine:	23 (25.3%)	20 (22.0%)	36 (39.6%)
Diazepam:	0	1 (1.1%)	13 (14.3%)

8. Do you use combined analgesia and sedation for your patients? (please tick one box)

Never	3 (3.3%)
Rarely	14 (15.4%)
Occasionally	26 (28.6%)
Frequently	21 (23.1%)
Very frequently	27 (29.7%)

9. Do you use continuous infusion of neuromuscular blocking agent for sedated and ventilated patients? (please tick one box)

Never	28 (30.8%)
Rarely	57 (62.6%)
Occasionally	4 (4.4%)
Frequently	1 (1.1%)
Very frequently	1 (1.1%)
